# Long non-coding RNA ENST00000500843 is downregulated and promotes chemoresistance to paclitaxel in lung adenocarcinoma

**DOI:** 10.3892/ol.2019.10704

**Published:** 2019-08-02

**Authors:** Xin Tian, Song Gao, Yang Liu, Ying Xuan, Rong Wu, Zhenyong Zhang

**Affiliations:** Department of Medical Oncology, Shengjing Hospital of China Medical University, Shenyang, Liaoning 110004, P.R. China

**Keywords:** long non-coding RNAs, lung adenocarcinoma, chemoresistance

## Abstract

Adenocarcinoma is one of the most common pathological types of human lung cancer and has the highest incidence and mortality rates worldwide. Resistance to paclitaxel (PTX), the standard chemotherapy agent for treatment of lung adenocarcinoma, is a major clinical obstacle. Sensitive markers are urgently required for the diagnosis and characterization of lung cancer, as well as to manage drug resistance. Previous studies have described the activity of long non-coding RNAs (lncRNAs) in human lung cancer and chemotherapy resistance. In previous studies, lncRNA ENST00000500843 was identified to be downregulated in PTX-resistant A549 human lung cancer cells. However, the roles of this lncRNA in the development of lung adenocarcinoma and its mechanism in PTX resistance, to the best of our knowledge, have not been described. In the present study, 56 pairs of lung adenocarcinoma and normal adjacent tissue samples were collected. Reverse transcription-quantitative PCR revealed that the expression levels of lncRNA ENST00000500843 were lower in lung adenocarcinoma tissues and PTX-resistant A549 cells when compared with normal adjacent tissues and A549 cells. Decreased expression levels of lncRNA ENST00000500843 in lung adenocarcinoma tissues were associated with tumor diameter, the degree of pathological differentiation and metastasis of lymph nodes. Additionally, patients with low expression levels of ENST00000500843 exhibited poorer overall survival and progression-free survival rates. Furthermore, the present study demonstrated that knockdown of lncRNA ENST00000500843 with small interfering RNA decreased the likelihood of apoptosis in A549 cells and promoted resistance to PTX. This indicated that lncRNA ENST00000500843 may be a useful diagnostic marker of lung cancer and a good prognostic marker for resistance to treatment with PTX.

## Introduction

Lung cancer is one of the leading causes of cancer-associated mortality worldwide, and its incidence, along with aging populations and environmental pollution, is increasing (1). This is leading to a large social and economic burden, which seriously affects the development of health systems and the economy around the world (2). Adenocarcinoma is a major pathological cancer type, and molecular-based targeted therapy is required. Existing therapies include surgery, radiotherapy and chemotherapy with paclitaxel (PTX), all of which exhibit poor survival rates (3,4). Consequently, resistance to PTX is an obstacle for clinical treatment since it affects the curative effect and eventually leads to treatment failure (5). Drug resistance is divided into primary and secondary resistance (6). Previously, investigations into the mechanisms of drug resistance focused on proteins associated with the cell membrane, increased drug metabolism, alterations in cell cycle, cell apoptosis and autophagy disorders, and DNA damage repair ability (7,8). The mechanism behind PTX resistance is multifactorial and complex, and presents a significant challenge in the successful treatment of lung cancer (9). An improved understanding of the molecular mechanism underlying the development of this form of resistance is therefore required. The identification of molecular markers associated with PTX-associated tumor response could help to select patients who are likely to benefit from chemotherapy (10).

In previous years, evidence has increasingly indicated that long non-coding RNAs (lncRNAs) participate in drug resistance via multiple signaling pathways. Abnormal lncRNA expression serves an important role in the regulation of tumor cell apoptosis and drug resistance (11–14). For example, the long intergenic non-coding (LINC) RNA HOX transcript antisense RNA (HOTAIR) is expressed at significantly higher levels in A549 lung adenocarcinoma cells resistant to cisplatin (DDP) compared with A549 cells that are sensitive to DDP, *in vitro* knockdown of HOTAIR has been demonstrated to reduce DDP resistance in A549 human lung cancer cells (15). It has also been demonstrated that HOTAIR alters the cell cycle by downregulating the cyclin dependent kinase inhibitor 1A signaling pathway; and, therefore, reduces cell apoptosis and enhances drug resistance (15). As another example, lncRNA growth arrest specific 5 (GAS5) has been identified to be downregulated in lung cancer tissues compared with in adjacent normal tissues (16). In addition, the expression levels of lncRNA GAS5 are significantly downregulated in epidermal growth factor receptor (EGFR)-tyrosine kinase inhibitor (TKI)-resistant lung cancer cells compared with in EGFR-TKI sensitive cells, and *in vivo* and *in vitro* experiments demonstrated that GAS5 affects EGFR-TKI resistance by regulating the insulin like growth factor 1 receptor protein (16). As a final example, lncRNA maternally expressed 3 (MEG3), which is located on human chromosome 14q32, has previously been reported as a tumor suppressor (17). It is mainly expressed in normal human tissues, particularly in the brain and pituitary gland. MEG3 has been identified to be abnormally expressed in various types of human cancer, with increased expression regulating the proliferation and apoptosis of non-small cell lung cancer (NSCLC) by activating the p53 protein (18). Additionally, using lncRNA microarray and quantitative PCR (qPCR) assays, Liu *et al* (19) demonstrated that the expression levels of MEG3 are notably decreased in DDP-resistant A549/DDP cells compared with in sensitive cells. Further experiments demonstrated that MEG3 induces the mitochondrial apoptosis signaling pathway via p53 and Bcl-xL activation, leading to alterations in the sensitivity of A549 cells to DDP.

In a previous study, it was identified that lncRNA ENST00000500843 expression is downregulated in PTX-resistant A549 human lung cancer cells (20). The present study investigated the role of lncRNA ENST00000500843 in the development of lung adenocarcinoma and its clinical relevance, as well as its effect on PTX resistance. The results of the present study may provide novel molecular therapeutic targets, and may provide a novel direction to evaluate benefits from chemotherapy and reverse drug resistance in patients with lung adenocarcinoma.

## Materials and methods

### 

#### Clinical specimens

A total of 56 lung adenocarcinoma and normal adjacent tissue samples (>5 cm from the tumor) were collected at Shengjing Hospital of China Medical University between May 2014 and August 2016. Clinicopathological characteristics of the patients are presented in Table I. These patients included 23 men and 33 women with a median age of 58 years (range, 32–76 years). None of the patients had received any preoperative chemotherapy or radiotherapy, and none of the patients had a previous history of malignant disease. All tumorous tissue samples were diagnosed histopathologically by professional pathologists from Shengjing Hospital of China Medical University who were blinded to the present study. All specimens were obtained within 15 min after excision from the body and stored in liquid nitrogen prior to reverse transcription (RT)-qPCR. All patients were diagnosed by biopsy and no previous system treatment had been performed on the patients. All patients provided written informed consent and the present study was approved by the Ethics Committee of Shengjing Hospital of China Medical University.

#### Cell lines and culture

The A549 human lung adenocarcinoma cell line was purchased from the Type Culture Collection of the Chinese Academy of Sciences. A549/PTX cells were established in our laboratory in a previous study (20). Cells were cultured in RPMI-1640 medium supplemented with 10% FBS and 1% penicillin-streptomycin (all from Gibco; Thermo Fisher Scientific, Inc.) at 37°C in a humidified incubator with 5% CO_2_. The A549/PTX cell culture medium also contained 200 ng/ml PTX (Bristol-Myers Squibb) to maintain the drug-resistant phenotype. Cells in the logarithmic phase of growth were used in all experimental procedures.

#### RT-qPCR

Total RNA from frozen tissue specimens and cells was extracted using TRIzol^®^ reagent (Takara Bio, Inc.) according to the manufacturer's protocol. The concentration and purity of total RNA were measured on a Nanodrop spectrophotometer (Thermo Fisher Scientific, Inc.). RNA integrity was assessed by electrophoresis on a denaturing agarose gel (1%) followed by detection with ethidium bromide. Subsequently, total RNA was reverse transcribed using a PrimeScript RT reagent kit with genomic DNA Eraser (Perfect Real Time; Takara Bio, Inc.) according to the manufacturer's protocol. The reaction conditions of reverse transcription were: 42°C for 60 min, 99°C for 5 min and holding at 4°C. RT-qPCR analyses were conducted using the SYBR Green assay (Takara Bio, Inc.). Thermocycling conditions were as follows: One cycle of 95°C for 10 min, 95°C for 15 sec, followed by 40 cycles of 60°C for 1 min. lncRNA ENST00000500843 primers were designed using Primer (version 5.0; Premier, Inc.). All experiments were repeated three times. The primer sequences were: ENST00000500843 forward, 5′-CCTGGCTGAGGTGAATAA-3′ and reverse, 5′-TTGGACCCGAACATCTG-3′; GAPDH forward, 5′-TGCACCACCAACTGCTTAGC-3′ and reverse, 5′-GGCATGGACTGTGGTCATGAG-3′. The relative gene expression levels of ENST00000500843 were determined using the 2^−ΔΔCq^ method (21).

#### Cell transfection

Three small interfering RNAs (siRNAs) targeting lncRNA ENST00000500843 (siRNA-135, siRNA-197 and siRNA-7) were synthesized by the Jima Corporation (Shanghai GenePharma Co., Ltd.). The sequences of the siRNAs were as follows: siRNA-135 forward, 5′-CAAUUCUCAUCAUGUCAUAUC-3′ and reverse, 5′-UAUGACAUGAUGAGAAUUGCA-3′; siRNA-197 forward, 5′-CUUAUUGCUGAAUAUUGAACU-3′ and reverse, 5′-UUCAAUAUUCAGCAAUAAGUC-3′; siRNA-7 forward, 5′-GCUGAAUAUUGAACUUGAUCA-3′ and reverse, 5′-AUCAAGUUCAAUAUUCAGCAA-3′; negative control forward, 5′-UUCUCCGAACGUGUCACGUTT-3′ and reverse, 5′-ACGUGACACGUUCGGAGAATT-3′. All three siRNAs (10 nM) were transfected into A549 cells (1×10^5^ cells/well) using Lipofectamine^®^ 2000 (Invitrogen; Thermo Fisher Scientific, Inc.) according to the manufacturer's protocol. The efficiency of knockdown was detected 36 h later using RT-qPCR analysis with the aforementioned methodology, and the most stable cells were selected for downstream processes. Subsequent experimentation was performed 36 h after transfection.

#### Chemoresistance assay

To evaluate differences in chemoresistance, A549 cells (5×10^3^/well) transfected with siRNA or negative control RNA were seeded in 96-well plates and incubated at 37°C for 36 h. Following initial incubation, 10 ng/ml PTX was added to the cells, which were further incubated at 37°C in a humidified atmosphere containing 5% CO_2_ for 24 h. Subsequently, 10 µl Cell Counting Kit-8 reagent (CCK-8; Dojindo Molecular Technologies, Inc.) was added to each well (10%), and cells were incubated at 37°C for another 4 h. The absorbance of each well was determined using an ELISA reader (NanoDrop ND-1000; Thermo Fisher Scientific, Inc.) at a wavelength of 450 nm. Three independent experiments were performed with five duplicate wells.

#### Cell apoptosis analysis

Cell apoptosis was detected by flow cytometry. The cells (5×10^3^ cells/well) were cultured at 37°C with 10 ng/ml PTX for 24 h after transfection. Cells were then double stained using an Annexin V-FITC apoptosis detection kit (BD Pharmingen; BD Biosciences) according to the manufacturer's protocol. Cells were analyzed using a flow cytometer and CellQuest software (version 3.0; BD Biosciences). Cells were classified as viable, dead, early apoptotic or late apoptotic. The percentages of early apoptotic and late apoptotic cells were calculated and compared.

#### Statistical analysis

Data were analyzed using SPSS version 18.0 (SPSS, Inc.) and GraphPad Prism version 5.0 (GraphPad Software, Inc.) software. The expression levels of lncRNA ENST00000500843 in lung adenocarcinoma tissues were categorized as low or high expression according to the mean value. Survival curves were estimated using the Kaplan-Meier method, and P-values were obtained using log-rank test. An independent samples t-test was used to compare the differences between two groups and one-way ANOVA with the Bonferroni test used as the post-hoc test using SPSS software (version 18.0; SPSS, Inc.). All experiments were performed at least three times, therefore the data were calculated as the mean ± standard deviation, and P<0.05 was considered to indicate a statistically significant difference.

## Results

### 

#### lncRNA ENST00000500843 is downregulated in lung adenocarcinoma tissues and A549/PTX cells

**Figure 1.**
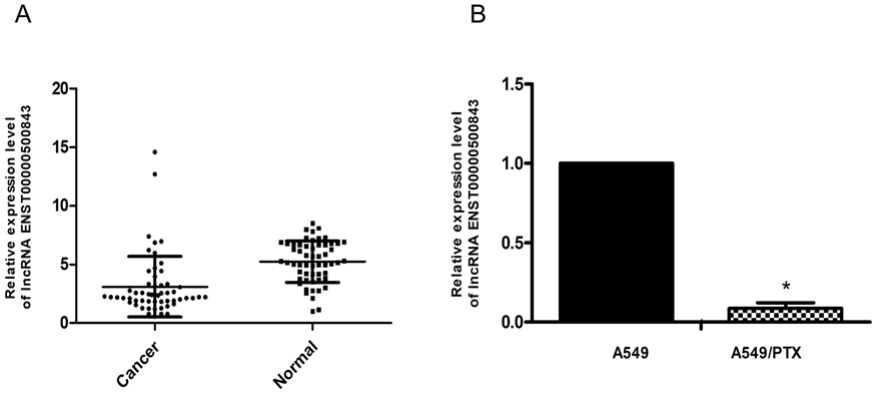
Expression levels of lncRNA ENST00000500843 in lung adenocarcinoma tissue and A549/PTX cells. (A) Expression levels of lncRNA ENST00000500843 were lower in lung adenocarcinoma tissues compared with in normal lung tissues. (B) Expression levels of lncRNA ENST00000500843 were lower in A549/PTX cells compared with in A549 cells. *P<0.05 vs. adjacent tissue or A549. A549/PTX, paclitaxel-resistant A549 cells; lncRNA, long non-coding RNA.

The present study compared the expression levels of lncRNA ENST00000500843 in lung adenocarcinoma and normal adjacent tissue samples. Total RNA was isolated from the tissue samples, and RT-qPCR analysis was performed to examine the expression levels of lncRNA ENST00000500843. The data demonstrated that the expression levels of lncRNA ENST00000500843 were markedly decreased in lung adenocarcinoma tissues compared with in normal adjacent tissues (P<0.05; Fig. 1A). Additionally, the present study examined lncRNA ENST00000500843 expression in A549/PTX and normal lung adenocarcinoma A549 cell lines. As presented in Fig. 1B, RT-qPCR analysis indicated that the expression levels of lncRNA ENST00000500843 were significantly decreased in A549/PTX cells compared with in A549 cells.

#### lncRNA ENST00000500843 expression is associated with clinicopathological characteristics

To further investigate the clinical significance of lncRNA ENST00000500843 in lung adenocarcinoma, the association between its expression and the clinicopathological characteristics of patients with lung adenocarcinoma was evaluated. In relation to the mean value (5.24), the 56 patients were divided into lncRNA ENST00000500843 high (n=11) and low (n=45) expression groups. The present study revealed that the expression levels of lncRNA ENST00000500843 in lung adenocarcinoma tissue were associated with tumor diameter, the degree of pathological differentiation and metastasis of lymph nodes (Table I; P<0.05). However, no association was identified between lncRNA ENST00000500843 expression and patient sex, age, smoking status and TNM stage (P>0.05; Table I) (22). These findings suggested that the decreased expression level of lncRNA ENST00000500843 is involved in the malignant progression of lung adenocarcinoma.

#### Downregulation of lncRNA ENST00000500843 is associated with poor prognosis

**Figure 2.**
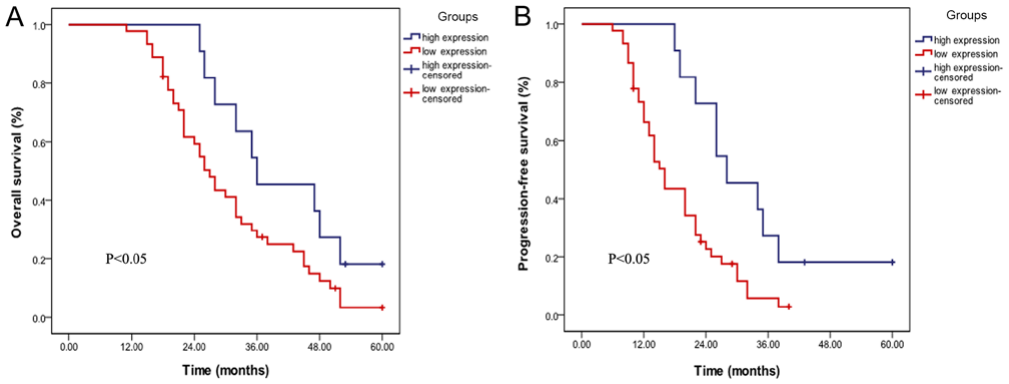
Association between lncRNA ENST00000500843 and progression of lung adenocarcinoma. (A) Patients with low expression levels of lncRNA ENST00000500843 exhibited poorer overall survival rates. (B) Patients with low expression levels of lncRNA ENST00000500843 exhibited poorer progression-free survival rates. lncRNA, long non-coding RNA.

The present study further analyzed the association between lncRNA ENST00000500843 expression and the survival time of patients with lung adenocarcinoma. Kaplan-Meier survival analysis was performed to estimate the association between lncRNA ENST00000500843 expression and the prognosis of patients with lung adenocarcinoma. It was demonstrated that patients with low expression levels of ENST00000500843 exhibited significantly poorer overall survival (OS; P<0.05; Fig. 2A) and progression-free survival (PFS; P<0.05; Fig. 2B) rates. The data suggested that patients with lung adenocarcinoma with lower lncRNA ENST00000500843 expression exhibited a worse prognosis compared with those with high expression levels of lncRNA ENST00000500843.

#### siRNA significantly decreases the expression levels of lncRNA ENST00000500843

**Figure 3.**
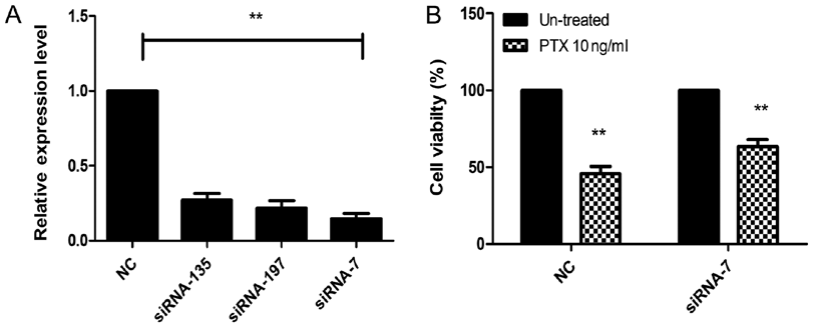
Knockdown of lncRNA ENST00000500843 increased chemoresistance to PTX in A549 cells. (A) After 36 h of transfection, relative expression levels of ENST00000500843 in A549 cells were measured by reverse transcription-quantitative PCR. Transfection of siRNA-135, siRNA-197 and siRNA-7 targeting ENST00000500843 significantly downregulated lncRNA ENST00000500843 expression in A549 cells, and siRNA-7 exhibited the most marked transfection efficiency. (B) Following transfection of siRNA-7, A549 cells were exposed to 10 ng/ml PTX for 24 h and cell viability was assessed by a Cell Counting kit-8 assay. Downregulation of ENST00000500843 increased resistance to PTX. **P<0.01 vs. si-NC; lncRNA, long non-coding RNA; NC, negative control; PTX, paclitaxel; siRNA, small interfering RNA.

In order to further study the association between ENST00000500843 and PTX resistance, siRNA-135, siRNA-197 and siRNA-7 were synthesized. As shown in Fig. 3A, compared with the negative control, all three siRNAs inhibited ENST00000500843 expression to varying degrees, with the strongest inhibition being observed for siRNA-7. The results suggested that ENST00000500843 expression was significantly downregulated following successful siRNA-7 transfection. Therefore, siRNA-7 was used for subsequent experiments.

#### Knockdown of lncRNA ENST00000500843 promotes chemoresistance to PTX in A549 cells

In order to investigate whether downregulation of ENST00000500843 impaired the resistance of A549 cells to PTX, A549 cells transfected with either siRNA-7 or control RNA were exposed to 10 ng/ml PTX for 24 h. Cell viability was calculated using the CCK-8 cytotoxicity assay. Compared with ENST00000500843 control cells, A549-siRNA knockdown cells exhibited significantly higher viability in a 10 ng/ml PTX atmosphere and, thus, less sensitivity to PTX (Fig. 3B). The results indicated that downregulated expression of lncRNA ENST00000500843 may promote PTX resistance in A549 cells.

#### Knockdown of lncRNA ENST00000500843 results in decreased levels of apoptosis in A549 cells and promotes lung adenocarcinoma resistance to PTX

**Figure 4.**
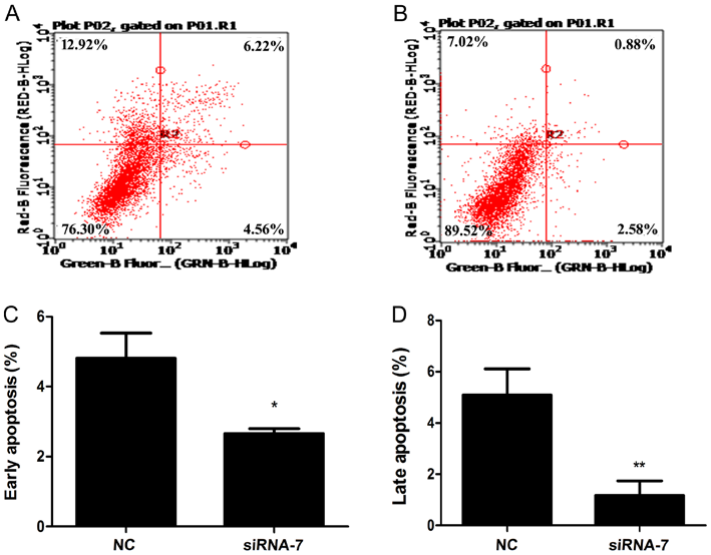
Knockdown of lncRNA ENST00000500843 decreases apoptosis in A549 cells and promotes resistance of lung adenocarcinoma cells to PTX. (A) Flow cytometric analysis of apoptosis in the NC group. (B) Flow cytometric analysis of apoptosis in transfection group. (C) Early apoptosis rates of NC and transfection groups when treated with 10 ng/ml PTX. (D) Late apoptosis rates of NC and transfection groups when treated with 10 ng/ml PTX. *P<0.05 and **P<0.01 vs. NC lncRNA, long non-coding RNA; NC, negative control; PTX, paclitaxel; siRNA, small interfering RNA.

To identify the mechanisms by which downregulated ENST00000500843 expression could contribute to PTX resistance, the present study assessed the effect that siRNA-induced knockdown of ENST00000500843 had on cell apoptosis. Following incubation with 10 ng/ml PTX, the results revealed that siRNA-7 bound to ENST00000500843 decreased the percentage of cells undergoing early and late apoptosis compared with the control siRNA (Fig. 4). Therefore, lncRNA ENST00000500843 could functionally modulate PTX-associated apoptosis and cell survival in A549 cells.

## Discussion

The present study demonstrated that the expression levels of lncRNA ENST00000500843 were significantly downregulated in lung adenocarcinoma tissues and A549/PTX cells compared with normal adjacent tissues and A549 cells. Additionally, it was demonstrated that the expression levels of lncRNA ENST00000500843 in lung adenocarcinoma were associated with tumor diameter, the degree of pathological differentiation, metastasis of lymph nodes and prognosis. Further experiments revealed that downregulation of lncRNA ENST00000500843 decreased the rate of apoptosis in A549 cells exposed to PTX, which led to the resistance of lung adenocarcinoma cells to PTX. The present study investigated the role of lncRNA ENST00000500843 in the development of lung adenocarcinoma and its clinical relevance, as well as its effect on PTX resistance.

lncRNA ENST00000500843 is located on human chromosome 8, 737 bp long and is categorized as belonging to the long intergenic non-coding RNA family (ENSEMBL; http://asia.ensembl.org/Homo_sapiens/Transcript/Summary?db=core;g=ENSG00000247134; r=8:32996178-33044855;t=ENST00000500843). To the best of our knowledge, there are no previous reports regarding its molecular mechanisms and clinical significance in lung adenocarcinoma and PTX resistance. In a previous study examining the microarray expression profile of lncRNAs in PTX-resistant A549 cells, it was identified that lncRNA ENST00000500843 expression was significantly downregulated in A549/PTX cells (20). Additionally, the results of the present study demonstrated that lncRNA ENST00000500843 expression was downregulated in lung adenocarcinoma tissues. The greater the downregulation of lncRNA ENST00000500843, the larger the diameter and the worse the pathological differentiation of the lung cancer. Lymphatic invasion was also frequently observed in such cases. This suggested that lncRNA ENST00000500843 may be involved in the incidence and development of lung cancer, and may function as a tumor suppressor. This hypothesis is supported by results from several other studies. For example, lncRNA-LINC00961 has been reported to be significantly downregulated in human NSCLC, and it could act as a tumor suppressor partially via affecting β-catenin expression (23). Zhang *et al* (24) demonstrated that lncRNA carbamoyl-phosphate synthase 1-HAUS augmin like complex subunit 3 expression is significantly decreased in colorectal carcinoma tissue and cell lines, and that it has a tumor-suppressive role in colorectal carcinoma.

As larger diameter and worse pathologic differentiation of tumors are generally associated with poorer prognosis of patients, one may speculate that lncRNA ENST00000500843 may affect the prognosis of patients with lung adenocarcinoma (25–28). The present study further investigated the association between ENST00000500843 expression and the OS and PFS rates of 56 patients. The data indicated that patients with low expression levels of ENST00000500843 had poorer OS and PFS rates compared with patients in the high expression group. This suggested that low expression levels of ENST00000500843 had a negative impact on the prognosis. Therefore, the clinical situation and survival of patients with lung adenocarcinoma may be predicted by studying the expression levels of ENST00000500843, making this lncRNA a potential candidate biomarker for the prognosis of lung adenocarcinoma.

Additionally, knockdown of lncRNA ENST00000500843 by siRNA-7 in A549 cells could inhibit cell apoptosis when treated with 10 ng/ml PTX, suggesting that, partly through the cell apoptosis signaling pathway, ENST00000500843 served a vital role in the regulation of PTX resistance. Overexpression of this lncRNA may result in increased sensitivity to PTX and provides a potential gene target for designing combination therapies with PTX. Although there are some lncRNAs which have been reported to be involved in PTX resistance of different types of cancer (29–32), the present study expanded the understanding of lncRNAs in the development and progression of lung adenocarcinoma and PTX resistance.

However, there were some limitations of the present study. Firstly, the present study focused on only one PTX-resistant lung adenocarcinoma cell line, and there is no data in PTX resistant tumor tissues, which made the experimental conclusions not sufficient. PTX resistant tumor tissues should be collected in future studies. Secondly, the present study only illustrated that the target lncRNA ENST00000500843 regulated PTX resistance in A549 cells via the cell apoptosis signaling pathway. However, the exact mechanisms by which lncRNA ENST00000500843 regulated the apoptosis signaling pathway are not known and require further investigation. Other experiments, including colony formation, spheroid generation and migration/invasion phenotype assays, should be performed in future studies.

In conclusion, the findings of the present study demonstrated that lncRNA ENST00000500843 was downregulated in human lung adenocarcinoma tissue and A549/PTX cells, may function as a tumor suppressor element, and that its expression was associated with tumor diameter, the degree of pathological differentiation and metastasis of lymph nodes. Additionally, patients with low expression levels of lncRNA ENST00000500843 exhibited poorer OS and PFS rates, implying that low expression levels of ENST00000500843 led to a worse prognosis. Additionally, the present study revealed that lncRNA ENST00000500843 partially regulated PTX resistance in A549 cells via the cell apoptosis signaling pathway. The present study improved the understanding of the function and clinical significance of lncRNA ENST00000500843 in the development of lung adenocarcinoma and PTX resistance, thereby establishing that lncRNA ENST00000500843 could be used as a novel marker to diagnose lung adenocarcinoma and evaluate the response to PTX-based chemotherapy. This could assist clinicians in identifying patients who would most likely benefit from chemotherapy. Future investigations into the molecular mechanisms and protein function are however required *in vivo* and *in vitro*.

## Figures and Tables

**Table I.** Association between lncRNA ENST00000500843 expression and clinicopathological features of patients with lung adenocarcinoma.

	lncRNA ENST00000500843		
		
Clinical characteristic	Low expression (n=45)	High expression (n=11)	P-value
Sex			
Male	19	4	0.990
Female	26	7	
Age, years			
<60	25	6	>0.999
≥60	20	5	
Smoking status			
Yes	24	7	0.781
No	21	4	
Tumor diameter, cm			
<2	13	8	0.019
≥2	32	3	
Pathological differentiation			
Moderate and low	36	4	0.012
High	9	7	
Metastasis of lymph nodes			
No	15	8	0.041
Yes	30	3	
TNM stage			
I, II	35	7	0.560
III, IV	10	4	

Independent Student's t-test and one-way ANOVA was used to compare the differences between two groups using SPSS software (version 18.0; SPSS, Inc.). Pathological differentiation was concluded by pathological pathologists from Shengjing Hospital of China Medical University using hematoxylin-eosin staining. TNM, Tumor-Node-Metastasis (22); lncRNA ENST00000500843, long noncoding RNA ENST00000500843.

## Data Availability

All data generated or analyzed during the present study are included in this published article.
